# Endoscopic ultrasound-guided radiofrequency ablation for large branch-duct intraductal papillary mucinous neoplasms: Safety and efficacy trial

**DOI:** 10.1055/a-2778-8145

**Published:** 2026-01-21

**Authors:** Somashekar G. Krishna, Erica Park, Jennifer Rath, Zarine Shah, Ahmed Abdelbaki, Stacey Culp, Fadi Hawa, Dan Jones, Wei Chen, Peter Lee, Hamza Shah, Jordan Burlen, Raj Shah, Mitchell L. Ramsey, Georgios I. Papachristou, Zobeida Cruz-Monserrate, Timothy Pawlik, Mary E Dillhoff, Jordan M. Cloyd, Susan Tsai, Phil A. Hart

**Affiliations:** 1 12306 Division of Gastroenterology, Hepatology and Nutrition, The Ohio State University Wexner Medical Center, Columbus, United States; 2 12306 Department of Radiology, The Ohio State University Wexner Medical Center, Columbus, United States; 3 12306 Department of Statistics and Biomedical Informatics, The Ohio State University Wexner Medical Center, Columbus, United States; 4 12306 Pathology, The Ohio State University Wexner Medical Center, Columbus, United States; 5 12306 Division of Surgical Oncology, The Ohio State University Wexner Medical Center, Columbus, United States

**Keywords:** Endoscopic ultrasonography, Pancreas, Intervention EUS, Endoscopy Upper GI Tract, RFA and ablative methods

## Abstract

**Background and study aims:**

Endoscopic ultrasound-guided radiofrequency ablation (EUS-RFA) is a nonsurgical treatment option for managing pancreatic lesions. We sought to evaluate the safety and efficacy of EUS-RFA for large (≥4 cm) branch-duct intraductal papillary mucinous neoplasms (BD-IPMNs).

**Patients and methods:**

Patients with a definitive diagnosis of BD-IPMN who declined or were unfit for surgery underwent EUS-RFA in a single-arm prospective trial. Ablation was performed using a 19G EUS-RFA needle. RFA applications were delivered up to a maximum threshold of 45 seconds or 400 ohms impedance. Safety was assessed using AGREE guidelines. Potential for efficacy was assessed using cyst volume and cyst fluid *KRAS GNAS* mutations using next-generation sequencing (NGS). Adverse events (AEs) were analyzed per RFA session, while response was analyzed per BD-IPMN.

**Results:**

Thirty BD-IPMNs (mean diameter 4.6 ± 1.7 cm; 80% multilocular) in 25 participants (mean age 74.1 ± 8.3 years) underwent 41 EUS-RFA sessions. AEs occurred in 12.2% of procedures (5/41), the majority being AGREE Grade 3A (9.8%, 4/41).

During a mean follow-up of 18 ± 5 months, 22 of 28 BD-IPMNs (78.6%) achieved ≥ 50% reduction in cyst volume, and 11 (39.3%) demonstrated complete (≥90%) response. Among 26 BD-IPMNs that revealed *KRAS GNAS* mutations, follow-up NGS was performed in 17, with 88.2% showing loss of detectable mutations.

**Conclusions:**

EUS-RFA in large, predominantly multilocular BD-IPMNs shows promising volumetric efficacy. Safety may be improved through refined energy delivery and technical advances. Molecular response remains exploratory and requires further validation. Long-term studies assessing progression-free outcomes are needed to define its role as an organ-preserving therapeutic option.

## Introduction

Management of branch duct intraductal papillary mucinous neoplasms (BD-IPMNs) relies on clinical and imaging criteria, including radiological and endoscopic ultrasound (EUS) findings, categorized as Kyoto “worrisome features” and “high-risk stigmata” (HRS) 1 . Surgery for BD-IPMNs is recommended when Kyoto-HRS or ≥3 worrisome features are present, indicating >65% neoplasia risk 1 2 . However, surgery carries substantial risks, with 1% to 3% mortality and 20% to 40% morbidity 3 . Moreover, over half of resected BD-IPMNs in expert centers reveal only low-grade dysplasia, and the increasing resections have not reduced invasive cancer rates, collectively suggesting surgical overtreatment of BD-IPMNs 1 4 .

EUS-guided ablation of BD-IPMN, serves as a minimally invasive option for patients who are unsuitable for surgery but have a reasonable life expectancy. As noted in prior position statements, EUS-chemoablation is limited by reduced efficacy in larger cysts due to inability to inject sufficient dosages of chemotherapeutic drug under US Food and Drug Administration dose restrictions, and it is generally not feasible for multilocular lesions 5 6 . EUS-guided radiofrequency ablation (RFA) provides an energy-based approach that may overcome these limitations by enabling thermal coagulation across complex cyst architecture. Early data suggest feasibility and potential efficacy, although prior studies were limited by small sample sizes, mixed pancreatic cystic lesion populations, and variable pre-ablation diagnostics 7 8 9 .

In this prospective, single-arm clinical trial, we evaluated EUS-RFA exclusively in large, predominantly multilocular BD-IPMNs. This study is distinguished by comprehensive pre-ablation characterization using needle-based confocal laser endomicroscopy (nCLE) and next-generation sequencing (NGS), combined with post-treatment assessment incorporating cyst-fluid molecular profiling and computerized three-dimensional (3D) volumetric analysis for objective response quantification.

## Patients and methods

Consecutive patients were enrolled to participate in a single-center clinical trial evaluating EUS-RFA for BD-IPMNs (the ERASE Study, NCT05961982) from May 2023 to November 2024. The study received approval from the institutional review board at The Ohio State University Wexner Medical Center (Study Number: 2023C0004). Informed consent and authorization for information release were obtained from all participants.

### Study design

**Fig. 1:**
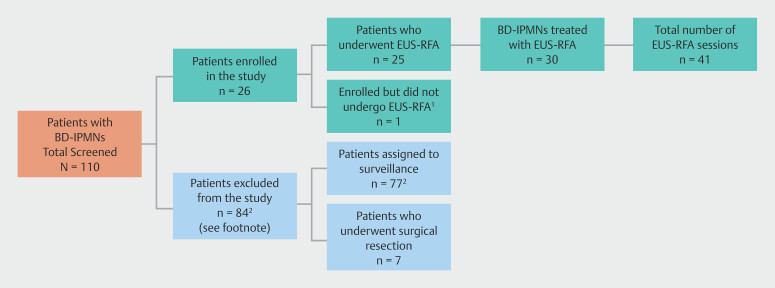
Study flow diagram. BD-IPMN, branch duct intraductal papillary mucinous neoplasm; EUS-RFA, endoscopic ultrasound-guided radiofrequency ablation (EUS-RFA). ^1^ BD-IPMN was not safely accessible due to intervening splenic vessels in the pancreatic tail. ^2^ Eighty-four were excluded for the following reasons: absence of high-risk or worrisome features (n = 62), Clinical Frailty Score (CFS) >6 (n = 14), surgical candidacy (n = 7), and a recent episode of acute pancreatitis (n = 1).

Only patients with a definitive diagnosis of BD-IPMN were included ( Fig. 1 , **Supplementary Fig. 1** ). Diagnostic criteria for BD-IPMNs included EUS- nCLE imaging demonstrating papillary structures or epithelial bands ( Video 1 ), or cyst-fluid NGS detecting *KRAS, BRAF* , or *GNAS* mutations and variants in other genes for risk stratification 1 . All BD-IPMNs underwent baseline NGS analysis and EUS-nCLE was performed in all but one BD-IPMN 10 11 . Baseline standard diagnostic cyst-fluid analyses (carcinoembryonic antigen, glucose, and cytology) were also performed. The primary objectives were to assess the efficacy and safety of EUS-RFA to treat BD-IPMNs. Assessment of long-term response to EUS-RFA was a secondary objective.

Eligible BD-IPMNs measured ≥ 3 cm incorporating at least one Kyoto-HRS or worrisome feature 1 . Pretreatment assessments included magnetic resonance imaging (MRI)/magnetic resonance cholangiopancreatography (MRCP) or pancreatic protocol computed tomography (CT) imaging, clinical frailty score (CFS), age-adjusted Charlson Comorbidity Index (CCI), EUS with nCLE, NGS, Kyoto criteria, and cyst-fluid analyses (CEA, glucose, cytology) ( **Supplementary Table 1** ) 12 13 . Patients were deemed non-operative candidates following surgical consultation and multidisciplinary tumor board review. Exclusion criteria included acute pancreatitis within 4 weeks of EUS-RFA, and pregnancy. All authors had full access to the study data and approved the final manuscript.

### EUS-RFA procedure

Endoscopic Ultrasound–Guided nCLE and Radiofrequency Ablation for Branch-Duct IPMN.Video 1

**Fig. 2:**
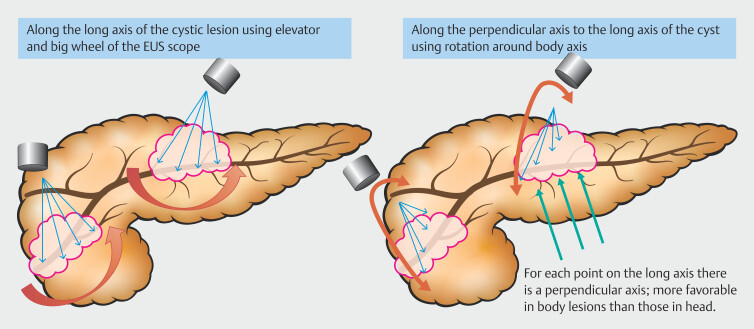
EUS-guided needle trajectory optimization for cystic lesions: longitudinal and perpendicular approaches. Schematic illustrating two approaches for EUS-RFA needle access in BD-IPMNs: (left) along the long axis using the elevator and big wheel of the EUS scope, and (right) along a perpendicular axis via scope rotation. For each point on the long axis, a corresponding perpendicular axis can be targeted, which is more feasible in body lesions than in those located in the pancreatic head or uncinate process.

A detailed audiovisual demonstration of the procedure is provided in Video 1 , with the EUS-RFA technique illustrated in Fig. 2 . EUS-FNA of the BD-IPMN was performed using a 19G or 22G FNA needle, aspirating most of the cyst fluid while leaving a small residual volume for RFA targeting. In multiloculated cysts, intracystic septations were punctured for complete aspiration. RFA was performed using the VIVA combo system (STARmed, Goyang, South Korea) with the 19G EUSRA 10 mm electrode (STARmed) at 50W in Continuance Mode, each application limited to 45 seconds or terminated at 400 Ohms. Multiple passes were permitted per lesion, with total cumulative ablation time per session defined as the sum across all applications.

Prophylaxis for post-EUS-RFA pancreatitis consisted of intravenous Ringer’s lactate (1–2 L) and intraprocedural rectal indomethacin, following the standard regimen established for endoscopic retrograde cholangiopancreatography (ERCP) prophylaxis 7 14 . Antibiotic prophylaxis consisted of intravenous ciprofloxacin 400 mg (or equivalent), followed by a 5-day oral course. Adverse events (AEs) were recorded using the AGREE classification, and pancreatitis severity per the Revised Atlanta Classification 15 16 .

The technique of EUS-RFA, as illustrated in the Fig. 2 , involves precise probe placement and lesion targeting using two primary axes of approach. The “long axis” approach involves positioning the probe along the cyst’s long axis using the elevator and large wheel of the EUS scope, enabling linear alignment and effective energy delivery. Ablation proceeds distally to proximally, with additional passes as needed for complete treatment. In the perpendicular axis approach, the probe is rotated (shaft or operator’s body axis) to achieve a perpendicular orientation relative to the cyst’s long axis. Strategic endoscope shaft rotation ensures optimal probe positioning, allowing thorough ablation at multiple points. Both approaches require precise manipulation of the EUS scope and probe. Excessive use of the elevator should be avoided to minimize mechanical stress and reduce risk of probe damage during lesion targeting and ablation.

BD-IPMNs were monitored per Kyoto guidelines with imaging every 3–6 months post-RFA ( **Supplementary Fig. 2** ) *.*

### Cyst-fluid molecular analysis

Pancreatic cyst-fluid NGS was performed on a same-day basis at the Ohio State University James Molecular Laboratory using a validated platform 10 . Total nucleic acid was extracted with the QIAamp UltraSens Virus Kit (QIAGEN). Samples with <5 ng/μL nucleic acid were concentrated using Microcon devices (Millipore/Merck). NGS utilized a PCR-based custom AmpliSeq assay on the Ion Chef and S5 platforms (Thermo Fisher, Waltham, Massachusetts, United States). Variants in 50 neoplasm-associated genes were analyzed using human genome build hg19, with variant calling via Torrent Suite and Genomic Oncology Software (Cleveland, Ohio, United States). The assay, validated for 0.5–2% variant sensitivity, achieved a mean read depth of ≥2000 reads in most samples 10 .

### Measurement of cyst volume

The process involved meticulous region of interest (ROI) marking to delineate cyst boundaries on each imaging slice (MRI or CT scan), followed by slice-by-slice ROI delineation. The selected ROIs were then used to calculate 3D volumes for each cyst using image viewer platform (Visage 7 Visage imaging Inc., San Diego, California, United States). This method improves accuracy and interobserver reliability over diameter-based measures, especially for BD-IPMNs, which are irregular and often fragment post-EUS-RFA due to intervening fibrosis 17 . Two dedicated radiologists (JR, ZKS) systematically reviewed all cross-sectional imaging studies, including surveillance scans, to generate image-based 3D volumetric data. To ensure consistency, all volumetric analyses adhered to standardized imaging protocols, and discrepancies between readers were resolved by consensus review in joint reading sessions.

### Outcome measures

The effectiveness of EUS-RFA was evaluated using volumetric and molecular response metrics.

#### Volumetric response

Computerized 3D volumetric analysis offers superior sensitivity particularly in detecting minimal residual volumes or cyst fragmentation, a common feature of pancreatic cystic lesion (PCL) ablation. Given the sensitivity of this technique, traditional high thresholds for complete volume response (> 95%) are not applicable 7 9 18 . A ≥90% volume reduction threshold, therefore, was adopted to reflect meaningful clinical response while accounting for detectable post-ablation residuals.

#### Molecular response (exploratory)

NGS was used to assess molecular response, defined as a >90% reduction in variant allele fraction (VAF) of both *KRAS* and *GNAS* mutations 19 . VAF represents the proportion of sequencing reads that contain a specific mutation relative to the total reads thereby quantifying the tumor DNA burden. A significant drop in VAF post-ablation reflects a reduction in neoplastic cellular content, providing a sensitive, quantitative marker of biological response.

### Retreatment

Retreatment during the study, guided by multidisciplinary tumor board discussions, was considered for BD-IPMNs that: 1) failed to achieve a partial volumetric response (≥50% reduction in cyst volume); 2) demonstrated persistent molecular alterations on post-ablation NGS, including VAF trends; or 3) were deemed appropriate based on patient-specific factors such as comorbidities, age, and expected progression-free survival. Retreatment procedures adhered to the same technical parameters as the index EUS-RFA, ensuring consistency in treatment protocols.

### Statistical analysis

Statistical analyses were performed using R version 4.1.2 (R Foundation for Statistical Computing, Vienna, Austria) and SPSS version 29.0 (IBM SPSS, Armonk, New York, United States). Analyses were conducted at the level most relevant to the outcome. AEs were analyzed per treatment session (n = 41) because these events are attributable to individual procedures, whereas neoplasm response was analyzed per BD-IPMN (n = 30). Patient-level data were also analyzed for demographics. Categorical variables were compared using chi-squared or Fisher’s exact tests. For continuous variables, Student’s two-sample *t* -tests were used when assumptions of normality and equal variance were satisfied (assessed by Shapiro-Wilk test, Q-Q plots, and Levene’s test). When variances were unequal, Welch’s *t* -test was applied; when normality was not met, the Mann-Whitney U test was used. Comparisons were performed for both index and cumulative EUS-RFA treatments to evaluate differences in clinical and procedural variables by response category. Receiver operating characteristic (ROC) analysis also was performed to explore thresholds of RFA duration in predicting volumetric response. For all tests, a two-sided *P* <0.05 was considered statistically significant. Analyses were based on complete cases, with no imputation for missing data.

## Results

### Baseline characteristics

The study included 25 participants with a mean age of 74.1 years (standard deviation [SD] 8.3) ( Table 1 ). The majority were men (72.0%), and four (16%) presented with attributable symptoms. Detailed participant data and specifics are provided in **Supplementary Table 1** *.* Characteristics of the 30 BD-IPMNs treated with EUS-RFA included mean cyst size of 4.6 cm (SD 1.7) ( Table 1 ; **Supplementary Fig. 1** ). The majority (80.0%) were multilocular and 70.0% were situated in the head/uncinate region of the pancreas. NGS revealed *KRAS* and/or *GNAS* mutations indicative of BD-IPMN in 86.7% of cases, with a mean VAF of 24.5% for *KRAS* and 16.6% for *GNAS* . Additional pathogenic variants implicated in progression, which were considered a high-risk feature, were found in 10.0% of cases ( Table 1 ). EUS-nCLE was performed in 29 BD-IPMNs and diagnostic papillary epithelium was visualized in all cases. Analysis based on the Kyoto criteria indicated that 6.7% had HRS, whereas 96.7% exhibited at least one worrisome feature. The most common worrisome features were cyst size ≥ 30 mm (93.3%) and a cyst growth rate of ≥5 mm over 2 years (50.0%).

**Table:** **Table 1** Characteristics of branch duct intraductal papillary mucinous neoplasms (BD-IPMNs) and technical features of endoscopic ultrasound-guided radiofrequency ablation (EUS-RFA) *.*

Participant characteristics	n *= 25 (%)*
**Demographics and referral information**	
Female (sex)	7 (28.0)
Age, years (mean, SD)	74.1 (8.3)
Race	
White	22 (88.0)
African-American	3 (12.0)
Asian	0 (0)
BMI, kg/m ^2^ (mean, SD)	28.8 (8.3)
History of diabetes mellitus	10 (40.0)
Clinical presentation (symptoms present)	4 (16.0)
Subjects who refused surgery	5 (20.0)
Clinical Frailty Scale (mean, SD)	3.4 (1.0)
Age-adjusted Charlson Comorbidity Index (mean, SD)	5.9 (1.7)
**Imaging/EUS data**	
Number of cysts observed:	
1	9 (36.0)
2 to 5	9 (36.0)
6+	7 (28.0)
**BD-IPMN characteristics (prior to treatment)**	***n = 30 (%)***
Cyst morphology	
Unilocular	6 (20.0)
Multilocular	24 (80.0)
Location	
Head/uncinate	21 (70.0)
Body/Tail	9 (30.0)
Size (maximum diameter), mean (SD) cm	4.6 (1.7)
3D-volume, mean (SD) mL	42.6 (63.9)
Cyst fluid CEA ≥ 192 ng/mL (n = 25) ^*^	11 (44)
Cyst fluid glucose ≤10 mg/dL (n = 26) ^†^	22 (84.6%)
Cyst fluid NGS ^‡^	
*KRAS* or *GNAS/BRAF* mutations	26 (86.7)
Variant allele fraction for *KRAS* , mean (SD) %	24.5 (18.6)
Variant allele fraction for *GNAS* , mean (SD) %	16.6 (19.5)
Presence of high risk mutations ^§^	3 (10.0)
**Kyoto variables**	
High-risk stigmata (any):	2 (6.7)
Obstructive jaundice	1 (3.3)
Solid or enhancing nodule (≥ 5 mm)	1 (3.3)
MPD dilated ≥ 10 mm	0 (0)
Suspicious or positive cytology	1 (3.3)
Worrisome feature (any):	29 (96.7)
Acute pancreatitis	1 (3.3)
Increased serum CA19–9 (> 37 U/mL)	2 (6.9)
New onset diabetes (past year)	3 (10.0)
Cyst size ≥ 30 mm	28 (93.3)
Enhancing nodule < 5 mm	0 (0)
Thick/enhancing cyst wall	7 (23.3)
MPD (5–9 mm)	4 (13.3)
Abrupt change in caliber of MPD with distal pancreatic atrophy	1 (3.3)
Lymphadenopathy	0 (0)
Cyst growth rate ≥ 5 mm/2 years ^§^	15 (50.0)
Worrisome feature (by count)	
≥1	29 (96.7%)
≥2	23 (76.7%)
≥3	7 (23.3%)
** Index EUS-RFA ^¶^**	***n = 30***
Cyst fluid volume aspirated prior to RFA, mL (mean, SD)	37.0 (73.6)
Cyst fluid volume aspirated (% of total IPMN volume)	63.9 (31.2)
Route	
Transgastric	16 (53.3)
Transduodenal	14 (46.7)
Total number of RFA applications (mean, SD)	14.4 (8.3)
Percentage of RFA applications reaching impedance (mean, SD)	69.7 (36.4)
Total time of RFA application, seconds (mean, SD) ^††^	313.7 (300.2)
Total duration of follow up, months (mean, SD)	18 (5.0)
** Cumulative EUS-RFA procedures ^‡‡^**	**n = 41**
Number of BD-IPMNs Requiring a Second EUS-RFA Procedure	10
Cyst fluid volume aspirated prior to RFA, mL (mean, SD)	45.5 (87.6)
Cyst fluid volume aspirated (% of total IPMN volume)	62.9 (30.8)
Route	
Transgastric	18 (43.9)
Transduodenal	23 (56.1)
Total number of RFA applications (mean, SD)	15.6 (9.5)
Percentage of RFA applications reaching impedance (mean, SD)	62.9 (39.9)
Total time of RFA application, seconds (mean, SD)	326.3 (285.4)
*The cyst fluid exhibited notable viscosity in certain cases of BD-IPMNs, thereby hindering the laboratory's ability to analyze CEA and/or glucose. ^†^ The 2024 Kyoto guidelines required any of the following high-risk stigmata on MRI or EUS: 1) obstructive jaundice; 2) enhancing solid component or mural nodule ≥ 5 mm (including intraductal); 3) main pancreatic duct ≥ 10 mm; or 4) suspicious or positive cytology for malignancy. ^‡^ Next-generation sequencing analysis. ^§^ High-risk mutations on NGS include *TP53, PIK3CA, SMAD4, PTEN, CDKN2A* , and *AKT1* . ^¶^ The 2017 Revised Fukuoka Criteria were applied because data collection began before the release of the 2024 Kyoto guidelines. **The initial EUS-RFA procedure performed for the BD-IPMN. ^††^ If impedance (> 400 ohms) was not reached, the RFA was stopped at a maximum of 45 seconds. ^‡‡^ The total number of EUS-RFA procedures performed for the BD-IPMN, including second and third treatments. BMI, body mass index; EUS, endoscopic ultrasound; FNA, fine needle aspiration; RFA, radiofrequency application; SD, standard deviation.	

### EUS-RFA procedure parameters

#### Index EUS-RFA procedure

For the index EUS-RFA procedures, mean cyst-fluid volume aspirated immediately prior to RFA was 64% of the total IPMN volume ( Table 1 ). A mean of 14.4 (SD 8.3) RFA applications were performed per procedure, with 70% of these applications achieving a preset maximum impedance of approximately 400 Ohms. Mean total RFA application time, calculated as the product of the total number of applications and duration of each application, was 5.2 minutes (SD 5.0) per BD-IPMN. Post-RFA follow-up imaging was available in 28 of 30 BD-IPMNs with a mean follow-up of 18 months (SD 5.0).

#### Cumulative EUS-RFA procedures

In 30 BD-IPMNs, nine cysts were treated with additional EUS-RFA sessions. Specifically, eight cysts underwent two treatment sessions, whereas one cyst required three sessions. This resulted in a cumulative 41 EUS-RFA procedures across the cohort as shown in Table 1 . Considering all procedures, the average number of RFA applications per session was 15.6 (SD 9.5), with 63% reaching maximum preset impedance. Mean total RFA application time per procedure was 5.4 minutes (SD 4.8).

### Treatment response

**Fig. 3:**
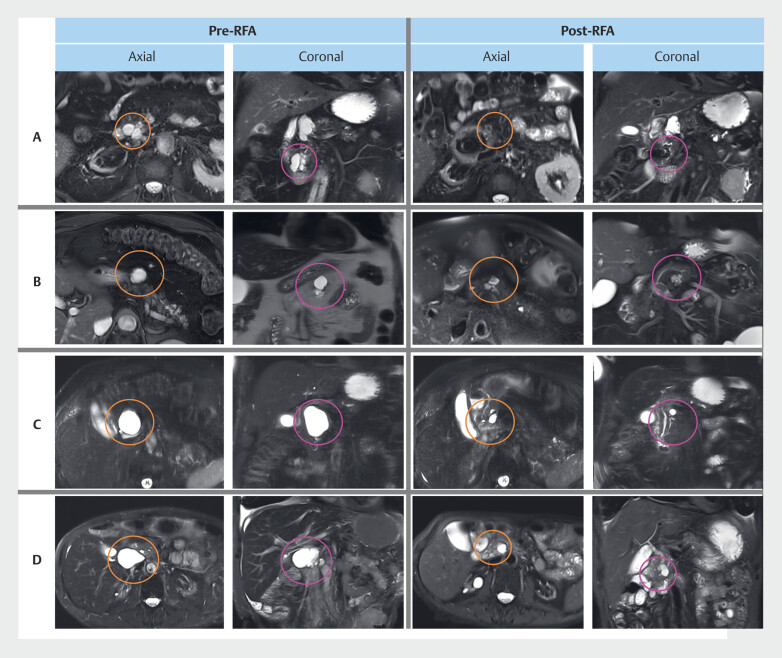
Representative volume reduction of BD-IPMNs assessed with MRI following endoscopic ultrasound-guided radiofrequency ablation (EUS-RFA). Each row represents a single BD-IPMN from a unique participant, with the lesion highlighted in axial images (orange circle) and coronal images (pink circle). BD-IPMN volumes were quantified using region-of-interest (ROI) segmentation and computerized three-dimensional (3D) reconstruction. The respective post-RFA volume reductions for BD-IPMNs in Panels A, B, C, and D, are 97%, 54%, 94%, and 99%, respectively.

Volumetric response: During a mean follow-up of 18 ± 5.0 months, 78.6% (22/28) of BD-IPMNs showed partial (≥50%) volume reduction, with 39.3% (11/28) achieving complete (≥90%) volume response ( Table 2 , Fig. 3 ).

**Table:** **Table 2** Evaluation criteria and response rates of branch duct intraductal papillary mucinous neoplasms (BD-IPMNs) following endoscopic ultrasound-guided radiofrequency ablation (EUS-RFA).

BD-IPMN parameters	Criteria	Response type	Rate of response
**Changes in dimensions**			
Volume (n = 28) ^*^	≥ 90% decrease in cyst volume	Complete	11 (39.3%)
Volume (n = 28)	≥ 50% decrease in cyst volume	Partial	22 (78.6%)
	< 50% decrease in cyst volume	Suboptimal	8 (21.4%)
**Change in molecular markers (NGS)**			
Diagnostic Mutations ^†^ (baseline n = 26; follow up NGS, n = 17)	Absence of *KRAS, BRAF* or *GNAS* mutations OR >90% decrease in VAF% of both *KRAS* and *GNAS* mutations	Complete	15 (88.2%)
High-risk mutations ^‡^ (n = 3)	Absence of detectable high-risk mutations	Complete	3 (100%)
*Of the 30 enrolled BD-IPMNs, two were excluded from follow-up: one elected hospice care due to comorbidities and complications, and another died from an unrelated cause prior to follow-up imaging after EUS-RFA. ^†^ Twenty-six were KRAS/GNAS positive at baseline. Of those, we have follow-up information for 17. Fifteen of 17 (88.2%) had a complete response. ^‡^ High-risk mutations: TP53, PIK3CA, SMAD4, PTEN, CDKN2A, and AKT1. BD-IPMN, branch duct intraductal papillary neoplasm; EUS-RFA, endoscopic ultrasound-guided radiofrequency ablation; NGS, next generation sequencing.			

Molecular response: Follow-up cyst aspiration with NGS was conducted specifically in those lacking complete volumetric response, demonstrating a molecular response in 88.2% of cases (15/17) ( Table 2 ). All three cases with high-risk mutations at baseline displayed negative NGS studies after treatment. A different *KRAS* mutation was detected in three BD-IPMN lesions, all of which were found in participants with multiple BD-IPMNs.

### Predictors of response

#### Index EUS-RFA procedure

After index EUS-RFA (n = 30 procedures), a comparison of BD-IPMNs with complete volume responses vs those without did not identify any patient- or cyst-related characteristics associated with a response ( Table 3 ). There was a significantly higher median cumulative RFA application time in the volume responder vs. non-responder groups (5.40 vs. 2.77 minutes, *P* = 0.02). Area under the ROC analysis identified an optimal RFA duration threshold of 201.5 seconds (3.36 minutes) for predicting complete volume response (sensitivity 88.9%, specificity 68.4%; **Supplementary Fig. 3** ).

**Table:** **Table 3** Comparison of BD-IPMN characteristics and EUS-RFA parameters for predictors of complete volume response.

	** Volume Response ** Index EUS-RFA			** Volume Response ** Cumulative EUS-RFA		
	** <90% ** (N = 19)	**≥90%** (N = 9)	*P* value	**<90%** (N = 17)	**≥90%** (N = 11)	*P* value
**Categorical variables**						
**BD-IPMN characteristics**						
Sex			1.000			1.000
Female (vs. male)	4 (66.7)	2 (33.3)		4 (66.7)	2 (33.3)	
Cyst morphology			0.352			0.022
Multilocular (vs. unilocular)	16 (72.7)	6 (27.3)		16 (72.7)	6 (27.3)	
Location			0.407			1.000
Head/uncinate (vs. body/tail)	14 (73.7)	5 (26.3)		12 (63.2)	7 (36.8)	
Viscosity of cyst fluid						0.671
Viscous (vs. non-viscous)	14 (70.0)	6 (30.0)		13 (65.0)	7 (35.0)	
Associated dilation of main pancreatic duct			1.000			1.000
Yes	3 (75.0)	1 (25.0)		3 (75.0)	1 (25.0)	
**Kyoto criteria**						
High risk stigmata (any) ^*^			1.000			1.000
Yes	1 (100)	0 (0)		1 (100)	0 (0)	
Presence of ≥ 3 Kyoto worrisome features ^†^			0.630			0.355
Yes	5 (83.3)	1 (16.7)		5 (83.3)	1 (16.7)	
**Continuous variables**						
Age	73.6 (8.3)	72.9 (7.0)	0.819	73.4 (8.8)	73.4 (6.4)	0.988
Body mass index	29.7 (6.8)	29.5 (4.9)	0.922	29.7 (7.2)	29.5 (4.5)	0.926
Maximum diameter of cyst, mean (SD), cm	5.0 (1.8)	4.0 (1.2)	0.175	4.9 (1.9)	4.4 (1.4)	0.461
Volume of cyst ^‡^ , mean (SD) ml	53.5 (77.8)	26.1 (19.8)	0.313	49.9 (80.3)	36.6 (35.2)	0.612
Volume of cyst ^§^ , median (IQR) ml	22.1 (36.6)	17.7 (21.5)	0.809	18.9 (34.2)	25.5 (27.9)	0.611
Cyst fluid volume aspirated (% of total IPMN volume) ^§^	69.5 (30.4)	56.9 (34.4)	0.335	62.2 (24.3)	61.7 (32.8)	0.969
Total number of RFA applications (mean, SD) ^§^	12.7 (8.9)	16.4 (7.2)	0.285	13.6 (8.1)	17.0 (7.0)	0.270
Total duration of RFA application, seconds (mean, SD) ^§^	248.5 (310.5)	462.4 (271.8)	0.089	220.8 (190.3)	453.0 (245.2)	0.009
Total duration of RFA application, seconds (median, IQR) ^§^	166.0 (183.0)	324.0 (484.5)	0.022	155.0 (151.5)	356.0 (442.0)	0.008
Percentage of RFA applications reaching impedance (mean, SD) ^§^	72.0 (37.7)	58.2 (34.8)	0.361	69.6 (30.7)	56.7 (31.4)	0.291
*Kyoto high-risk stigmata: obstructive jaundice, solid or enhancing nodule (≥5 mm), main pancreatic duct dilated ≥10mm, suspicious or positive cytology. ^†^ Kyoto worrisome features: History of acute pancreatitis, increased serum CA19–9 (>37 U/mL), new onset diabetes (past year), cyst size ≥30 mm, enhancing nodule <5 mm, thick/enhancing cyst wall, MPD (5–9 mm), abrupt change in caliber of MPD with distal pancreatic atrophy, lymphadenopathy, and cyst growth rate ≥5 mm/2 years. ^‡^ Volume - calculated by region of interest marking and computerized three-dimensional reconstruction. ^§^ For BD-IPMNs that underwent multiple EUS-RFA procedures, the mean was calculated across all RFAs performed. BD-IPMN, branch duct intraductal papillary neoplasm; EUS-RFA, endoscopic ultrasound-guided radiofrequency ablation; SD, standard deviation.						

#### Cumulative EUS-RFA procedures

Mean interval between the first and second RFA among the subjects (8 BD-IPMNs with 2 RFAs, 1 BD-IPMN with 3 RFAs) who underwent a second treatment was approximately 8 months and 22 days. Comparing volume response after cumulative EUS-RFA procedures (41 total, Table 3 ) revealed that unilocular cysts achieved significantly higher complete volume response rates (5/6 lesions; 83.3%) compared with multilocular cysts (6/22 lesions; 27.3%); *P* = 0.022. BD-IPMNs with complete volume response had longer median RFA durations (5.9 vs. 2.6 minutes, *P* = 0.008).

### Adverse events

AEs occurred in 12.2% of EUS-RFA sessions (5 of 41) and in 20% of participants (5/25), all involving BD-IPMNs located in the head/uncinate process of the pancreas. All complications were successfully managed nonsurgically. Table 4 presents detailed information on AGREE Grade classifications, administered treatments, and recommended preventive measures to reduce risk of similar complications in future procedures.

**Table:** **Table 4** Adverse events during endoscopic ultrasound-guided radiofrequency ablation for branch duct-intraductal papillary mucinous neoplasms: management strategies and preventive measures.

BD-IPMN number ^*^	Adverse Event	AGREE Grade and Revised Atlanta	Treatment	Outcome and preventive measures
28	Acute pancreatitis and duodenal perforation were caused by thermal injury due to loss of needle insulation ( **Supplementary Fig. 4** ), resulting from the melting of the active ice bath used to regulate the RFA needle	AGREE Grade IIIA Severe acute pancreatitis	Local endoscopic closure of perforation with over the scope clip and endoscopic suturing and conservative management of acute pancreatitis	Outcome: No sequelae of duodenal perforation or acute pancreatitis; patient completely recovered Preventive measures: Ensure ice bath remains intact during the procedure by checking the status of the ice frequently, including during procedure time-outs
19	Dilation of the common bile duct due to biliary stricture with elevation of alkaline phosphatase	AGREE Grade IIIA	Treated with ERCP, biliary dilation, and covered metal stent placement.	Outcome: Resolution of biliary stricture was achieved after stent removal Preventive measures: During RFA, ensure safe distance from distal common bile duct to prevent inadvertent injury and stricture
9	Duodenal ulcer leading to bleeding and short stricture formation. Patient was on Apixaban.	AGREE Grade IIIA	Bleeding managed with proton pump inhibitor and endoscopic measures. Stricture was managed with dilation and placement of lumen-apposing metal stent (LAMS)	Outcome: Patient elected hospice care due to multiple comorbidities Preventive measures: Consider proton pump inhibitor prophylaxis for patients undergoing multiple RFA applications in a single session. Consider delay of restarting anticoagulation in select patients
21	Pseudoaneurysm of a branch of the gastroduodenal artery resulting in a localized hematoma	AGREE Grade IIIA	Treated with angiographic-guided embolization by interventional radiology	Outcome: Embolization fully resolved the clinical consequences of pseudoaneurysm Preventive measures: Recognize pseudoaneurysm as a rare but possible complication of RFA, as reported in other systems like pulmonary and cardiac RFAs
2	Acute pancreatitis (mild) Patient had a prior history of recurrent acute pancreatitis and ongoing tobacco and alcohol abuse	AGREE Grade I mild acute pancreatitis	Intravenous fluids and symptomatic management	Outcome: Resolved with overnight observation Preventive measures: Evaluate patient history of pancreatitis and counsel on abstaining from alcohol and tobacco prior to procedures
*Refer to **Supplementary Table 1** for detailed data corresponding to each BD-IPMN case number. BD-IPMN, branch duct intraductal papillary neoplasm; LAMS, lumen-apposing metal stent; RFA, radiofrequency ablation				

### Technical complications and device malfunctions

EUS-RFA needle malfunction was observed in three of 41 procedures (7.3%). In one case, the insulation jacket was sheared, likely due to excessive use of the EUS scope elevator ( **Supplementary Fig. 4** ). In a second case, charring and melting of the needle insulation were noted due to inadequate cooling from a melted ice bath, resulting in severe acute pancreatitis with duodenal perforation ( **Supplementary Fig. 5** , Table 4 ). In the third case, excessive bending of the needle impeded actuation within the target lesion; the damaged needle was replaced, and the procedure was completed successfully.

### Unrelated mortality

During the study period, four unrelated deaths occurred within the study population. None of the deaths were related to pancreatic disease or the study procedures. One patient transitioned to hospice care due to multiple comorbidity-related issues, two patients suffered fatal cardiorespiratory events, and another patient succumbed to complications from a separate underlying malignancy. Mean CFS score was significantly higher in patients who died compared with survivors (4.75 ± 0.5 vs. 3.10 ± 0.9; *P* = 0.002).

## Discussion

This prospective trial is the largest study to exclusively assess EUS-guided RFA in definitively diagnosed large BD-IPMNs with pre-ablation molecular and endomicroscopic characterization.

Approximately 80% of BD-IPMNs achieved partial and 40% achieved complete volumetric response, with most *KRAS/GNAS* -mutated cysts showing complete molecular response. Optimal ablation time averaged 3.4 minutes. AEs occurred in about 12% of sessions, predominantly AGREE Grade 3A, requiring endoscopic or radiologic management without surgery.

A recent review of EUS-RFA for PCLs reported a 23% AE rate across 48 RFA sessions, with 2% classified as AGREE III/IV events 20 . In comparison, our study showed a lower rate of 12.2% (5/41 sessions), but with 9.8% (4/41) classified as AGREE IIIa events 21 . Table 4 details the specific treatment and mitigation measures implemented to address these risks. Probe malfunction occurred in approximately 5% of procedures (2/41), primarily due to excessive use of the elevator. Because the EUS-RFA probe is a steel needle, it is susceptible to bending, and the insulation jacket can shear under mechanical stress ( **Supplementary Fig. 4** and **Supplementary Fig. 5** ). Hence, we modified our technique to minimize elevator use during probe manipulation, particularly in cases requiring extreme scope angulation. Instead, we employed alternative maneuvers, such as torquing the endoscope shaft or partial scope reduction, to optimize probe positioning. These adjustments effectively reduced risk of probe damage in subsequent cases while maintaining precise lesion targeting. Beyond technical refinements, continued innovation in probe design and further study of ablation parameters, including power settings, number of applications, and energy lockout thresholds (impedance and duration), will be essential to balance safety with therapeutic efficacy in future applications of EUS-RFA.

A recent meta-analysis of EUS-guided ablation for PCLs reported a 44% complete resolution rate (95% confidence interval 31%-57%) and 30% partial response rate (≥50% size reduction) at ≥12 months. Subgroup analysis showed significantly lower resolution rates with RFA alone (13%) compared with ethanol (32%) and ethanol-paclitaxel (70%); **Supplementary Table 2** 18 . Our study demonstrated improved outcomes compared with RFA-alone studies, achieving 39.3% complete and 78.6% partial volume responses in BD-IPMNs. These outcomes are consistent with Barthet et al.'s 70.5% significant response (BD-IPMN = 16; decrease > 50 % or complete resolution) rate and Younis et al.'s 50% complete resolution in BD-IPMNs (n = 4) at 1 year ( **Supplementary Table 3** ) 7 9 . Unlike prior trials, which treated smaller cysts (2.9–3.65 cm) using one to seven RFA applications, our protocol targeted larger BD-IPMNs (mean 4.6 cm) using more applications (mean 14.4) at 50 W, terminating at 400 Ohms or 45 seconds. The study employed a 400 ohms impedance threshold and 45-second maximum duration, representing a middle ground between prior protocols. Barthet et al. used lower thresholds (100 ohms) whereas studies on pancreatic neuroendocrine tumors used higher thresholds (500–600 ohms) 7 9 . This modification accounts for the fluid content of cystic lesions, which requires greater thermal energy compared with solid tumors 7 9 . Multilocular BD-IPMNs demonstrated lower complete response rates compared with unilocular cysts. This likely reflects the technical limitations of uniformly ablating multiple compartments. Our analysis showed that a cumulative RFA duration of 3.36 minutes ( **Supplementary Fig. 3** ), representing the sum of multiple applications (each terminated at 400 Ohms or 45 seconds), was associated with optimal complete response. The higher volumetric response rate compared with prior RFA studies may be attributed to thorough fluid aspiration and septal puncture enabling uniform energy distribution and use of 3D volumetric analysis providing superior measurement sensitivity.

This study employed a quantitative approach to cyst-fluid molecular changes demonstrating a high response rate (88.2%, 15/17 cases) evidenced by a >90% drop in VAF of both *KRAS* and *GNAS* mutations. All three BD-IPMNs harboring high-risk mutations at baseline showed complete resolution post-treatment. A new clonal *KRAS* mutation emerged in three BD-IPMN lesions in patients with multifocal disease, possibly due to ductal communication or aspiration of adjacent BD-IPMNs. Although reductions in VAFs are biologically consistent with decreased neoplastic DNA burden, these measures are not validated biomarkers of malignancy or treatment success. VAFs can also be affected by clonal heterogeneity, sampling variability, and analytic factors. Using VAF as a quantitative measure provided greater precision than prior studies that only reported mutation presence or absence 22 23 . Our findings revealed a complex relationship between volumetric and molecular responses to EUS-RFA ( **Supplementary Table 4** ). Discordance between volumetric and molecular responses, including persistent cysts with complete molecular clearance, suggests cyst volume alone is insufficient to define efficacy and may reflect pseudocyst-like changes after RFA. Thus, cyst-fluid NGS should be considered exploratory and adjunctive, with prospective studies needed to determine its correlation with histopathology and long-term outcomes.

Several limitations warrant consideration. Most BD-IPMNs in our study did not meet Kyoto-HRS, which generally warrant surgery; thus, our findings represent an exploratory trial of EUS-RFA in nonsurgical patients with multiple worrisome features. The occurrence of four deaths unrelated to EUS-RFA procedure and follow-up underscores the need for optimal patient selection balancing malignant risk against comorbidity and life expectancy. To address this, we incorporated age-adjusted CCI, CFS, and patient preference alongside Kyoto criteria to guide eligibility 1 12 13 . The study was conducted at a single high-volume center by an experienced operator, which may limit generalizability. Although this is the largest prospective study of EUS-RFA for BD-IPMNs to date, the sample size remains modest. Post-RFA NGS was performed selectively in incomplete responders because cyst fluid could not be obtained from completely resolved lesions. This introduces potential selection bias favoring persistent mutation detection. Given the small sample, clustering was not modeled, and analyses were performed at the RFA-session or BD-IPMN level. Radiologists were not blinded to patient identity during volumetric analysis, which could introduce minor observer bias in volumetric measurements. Given only 11 complete volumetric responses, the study lacked sufficient events per variable to permit multivariable logistic regression. To avoid model overfitting, only univariable analyses were performed. Advanced modeling should be pursued in larger, multicenter studies. Finally, molecular response assessment using cyst-fluid NGS is susceptible to analytic variability and should be considered exploratory and hypothesis-generating only.

## Conclusions

In conclusion, EUS-RFA can be applied to substantially larger and often multilocular BD-IPMNs than those typically treated in ethanol or chemoablation trials, demonstrating encouraging safety and efficacy. Unlike injection-based methods, RFA provides direct tissue coagulation, offering technical feasibility in complex cysts. Importantly, this exploratory trial incorporated volumetric and molecular endpoints, which may enhance sensitivity in assessing biological response. Nevertheless, the central uncertainty remains: the incomplete understanding of IPMN carcinogenesis, both within the treated cyst and elsewhere in the pancreas. Whether ablating precursor lesions alters long-term cancer risk is unknown, and the clinical significance of molecular response requires validation. Given the non-negligible risks of AEs and device malfunction, careful patient selection and procedure refinement remain critical. At present, EUS-RFA can be considered for patients unfit for surgery or with cyst characteristics limiting other ablation techniques, but its role in cancer prevention will require longer-term, multicenter studies.

## Supplementary Material

Supplementary Material Zusatzmaterial
