# Supplementary material for: Endoscopic ultrasound-guided radiofrequency ablation for large branch-duct intraductal papillary mucinous neoplasms: Safety and efficacy trial
Source: Endosc Int Open. 2026 Jan 21;14:a27788145. doi: 10.1055/a-2778-8145 (PMC12828972; doi:10.1055/a-2778-8145)
Supplement: Supplementary file 2 — Supplementary Material [file 10-1055-a-2778-8145_27803199.pdf]

Thieme

Supplementary material. Title: Endoscopic Ultrasound-guided Radiofrequency Ablation for Large Branch-duct Intraductal Papillary Mucinous Neoplasms: A Safety and Efficacy Trial

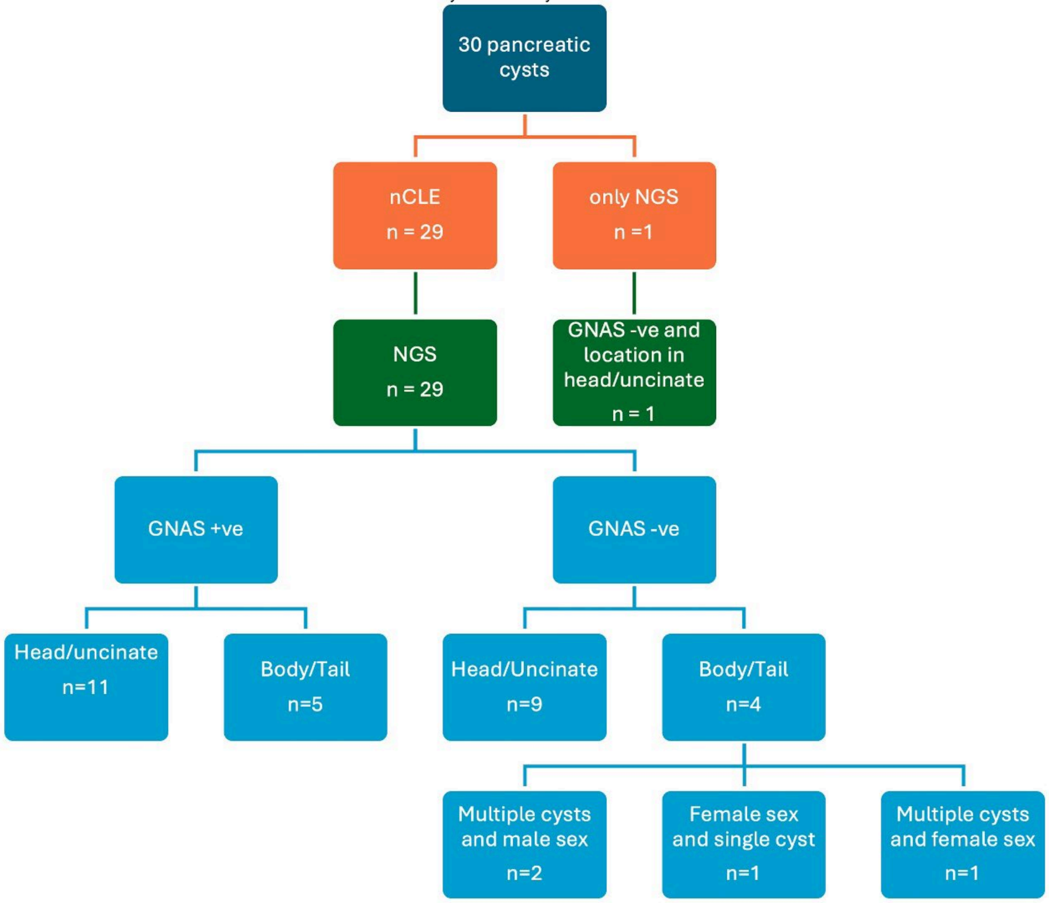

Supplementary Fig. 1 Diagnostic pathway for pancreatic cystic lesions (PCLs).

Diagnostic workflow and stratification of 30 pancreatic cysts evaluated using needle-based confocal laser endomicroscopy (nCLE) and next-generation sequencing (NGS). All nCLE exams (n = 29) demonstrated mucinous features, including characteristic epithelium or papillary fronds. Cysts were stratified by GNAS mutation status and anatomical location. GNAS mutations and head/uncinate location collectively with nCLE showing mucinous cyst are diagnostic for BD-IPMNs. GNAS-negative cysts were further evaluated based on cyst multiplicity and patient sex (male), as these features when combined with mucinous morphology on nCLE also support BD-IPMN diagnosis.

Endosc Int Open | DOI 10.1055/a-2778-8145 | © The Author(s).

Supplementary material. Title: Endoscopic Ultrasound-guided Radiofrequency Ablation for Large Branch-duct Intraductal Papillary Mucinous Neoplasms: A Safety and Efficacy Trial

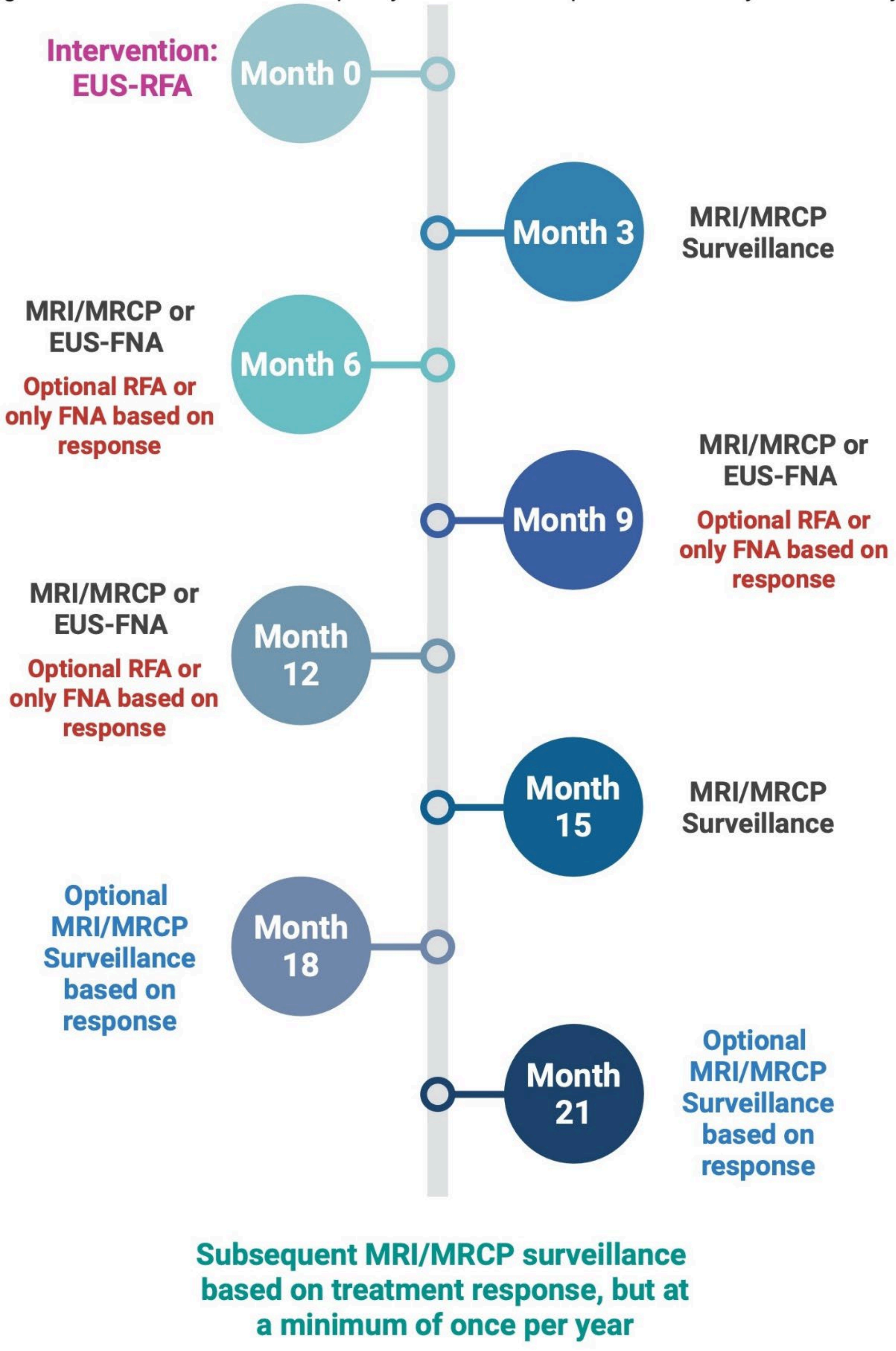

Supplementary Fig. 2 Study workflow: Schematic representation showing timepoints for EUS-RFA, follow-up imaging, and repeat EUS-RFA or EUS-FNA for molecular testing.

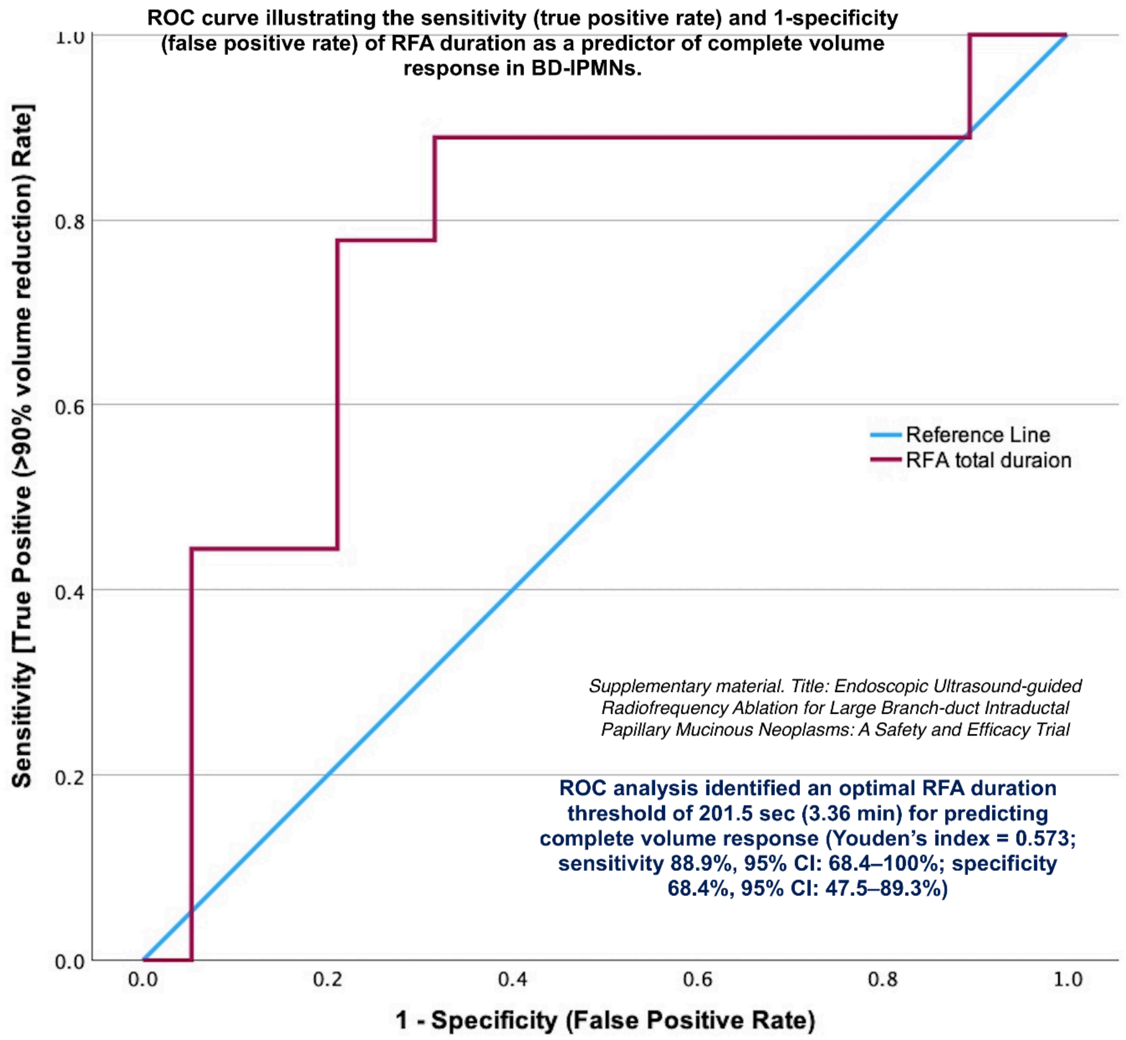

Supplementary Fig. 3 Receiver operating characteristic (ROC) curve illustrating the diagnostic performance of RFA duration in predicting complete volume response in BD-IPMNs. The curve plots sensitivity (true positive rate) against 1-specificity (false-positive rate) across a range of RFA duration thresholds. The area under the curve (AUC) quantifies the overall discriminative ability of RFA duration as a predictor, with higher values indicating better predictive accuracy. The optimal cutoff point, determined by the Youden Index, reflects the duration associated with the best balance between sensitivity and specificity for achieving complete cyst volume reduction.

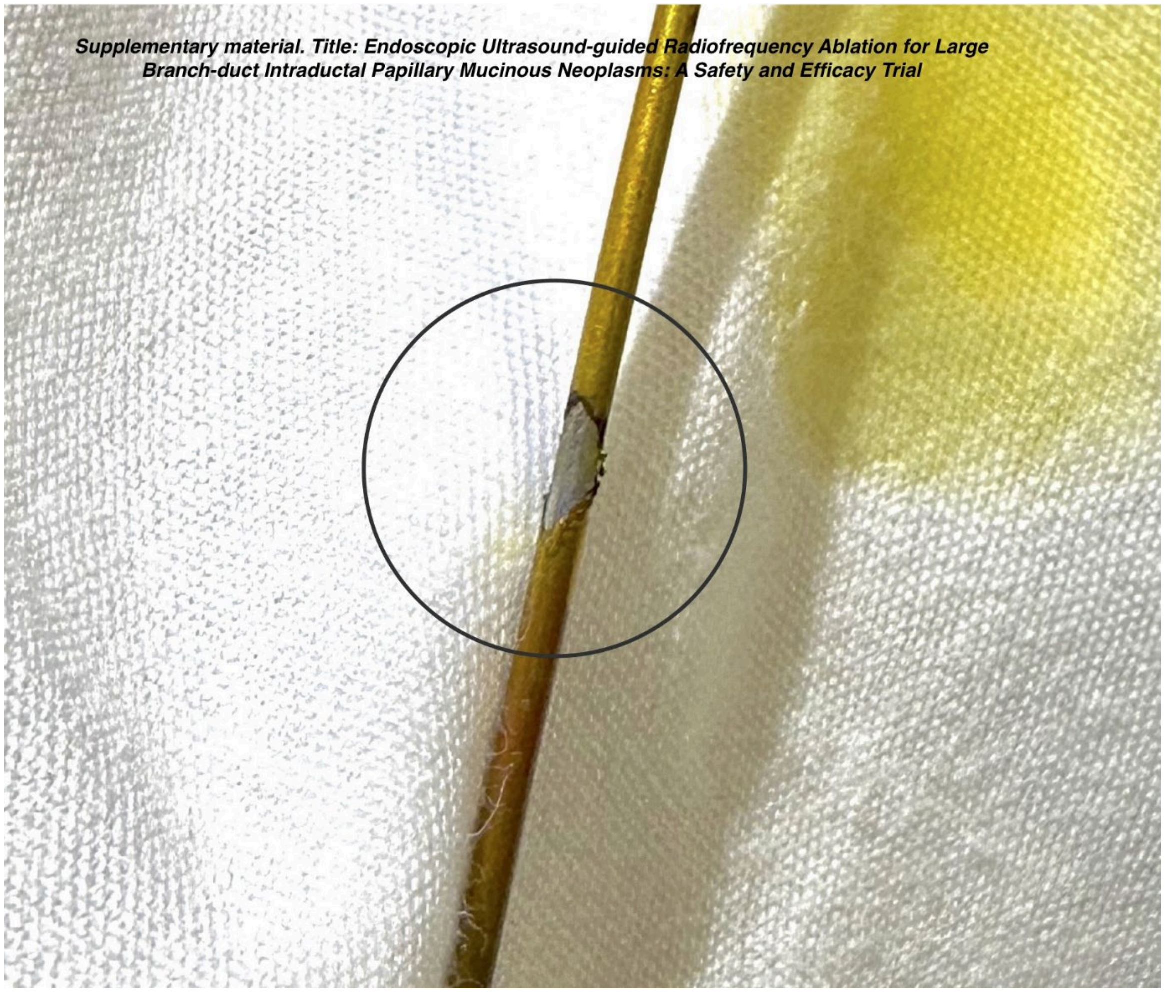

Supplementary Fig. 4 EUS-RFA needle insulation shearing due to excessive scope elevator use. Photograph demonstrating focal shearing of the EUS-RFA needle insulation jacket, likely resulting from repeated or excessive use of the EUS scope elevator during probe manipulation. No adverse events occurred in this case, but this highlights the importance of minimizing elevator use to prevent device damage.

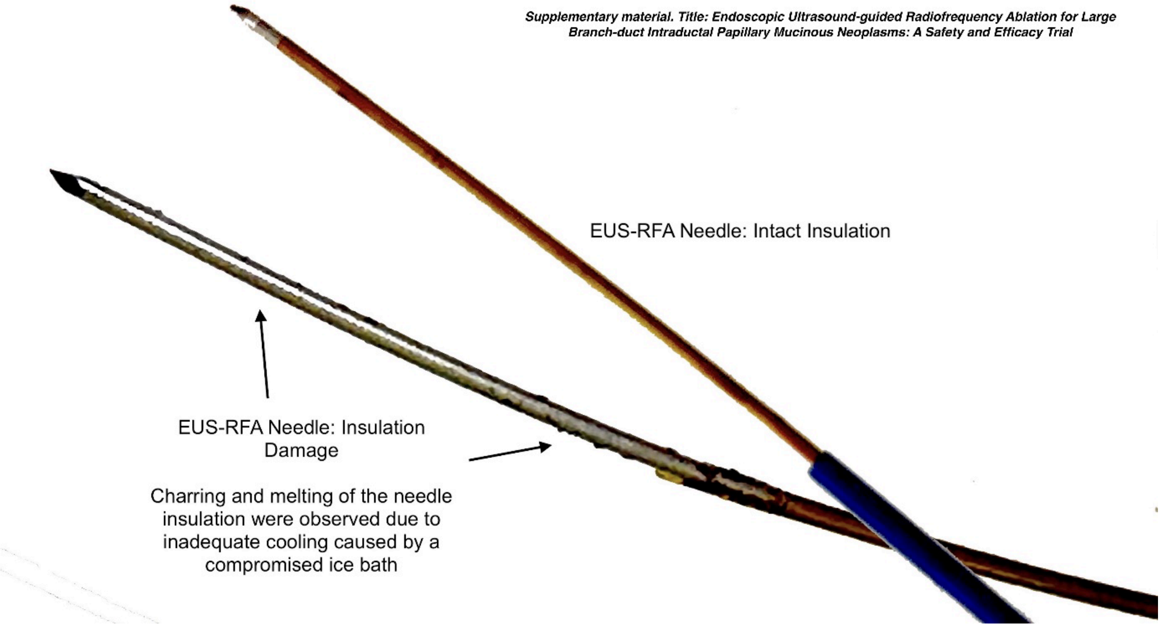

Supplementary Fig. 5 Charring and melting of EUS-RFA needle insulation from inadequate cooling. Footnote: Comparison of EUS-RFA needles showing insulation damage (lower) versus intact insulation (upper). Charring and melting of the needle insulation occurred due to inadequate cooling from a compromised ice bath, resulting in severe acute pancreatitis with duodenal perforation. Proper maintenance of the ice bath is critical to prevent thermal injury and device malfunction.

Supplementary Table 1 Participant-level data and intraductal papillary mucinous neoplasm (IPMN) characteristics.

											Kyoto High Risk Stigmata							Kyoto Worrisome Criteria													
BD-IPMN number	Age	Clinical Frailty Scale (CFS)	Charlson Comorbidity Index (age-adjusted)	Maximum dimension on EUS	Maximum dimension on cross-sectional imaging	Volume of BD-IPMN	Location of BD-IPMN	Locularity of BD-IPMN	Symptomatic due to BD-IPMN	Jaundice due to BD-IPMN	Presence of mural nodule ≥ 5 mm	Main pancreatic duct (MPD) dilation ≥ 10 mm	Positive cytology for HGD/ICa	Cyst size ≥ 30 mm	Enhancing cyst wall	MPD dilation 5-9.9 mm	Presence of mural nodule < 5 mm	Change in MPD caliber upstream of the cyst	Rapid cyst growth ≥ 5 mm/ year	Suspicious lymphadenopathy	Elevated serum CA 19-9 levels	New-onset diabetes mellitus	Acute pancreatitis	CEA	Glucose	*KRAS* variant allele fraction at baseline	*GNAS* variant allele fraction at baseline	Presence of high-risk DNA mutations	Total RFA duration per BD-IPMN	Number of RFA applicaations	Second EUS-RFA procedure
1	91.00	4.00	4	54	47.00	30.80	Body/tail	Multilocular	Incidental	No	No	No	No	Yes	No	No	No	No	No	No	No	No	No	24.6	<10	35.80	44.70	No	414.00	10.00	Yes
2	60.00	3.00	9	35	37.00	15.30	Head/uncinate	Multilocular	Incidental	No	No	No	No	Yes	Yes	Yes	No	No	No	No	No	No	No	442.1	<10	0.00	0.00	No	155.00	12.00	Yes
3	77.00	3.00	6	23	23.00	1.20	Head/uncinate	Multilocular	Symptomatic	No	No	No	No	No	Yes	No	No	No	No	No	No	Yes	No	8.0	<10	21.80	0.00	Yes	48.50	2.00	Yes
4	69.00	5.00	6	54	41.00	25.50	Head/uncinate	Multilocular	Incidental	No	No	No	No	Yes	No	No	No	No	No	No	No	No	No	<2.0	103	37.00	0.00	No	69.00	6.00	No
5	79.00	2.00	6	57	61.00	40.90	Body/tail	Multilocular	Incidental	No	No	No	No	Yes	No	Yes	No	No	Yes	No	Yes	No	No	62.5	<10	30.20	29.20	No	137.00	10.00	No
6	76.00	3.00	5	43	39.00	7.00	Head/uncinate	Multilocular	Incidental	No	No	No	No	Yes	Yes	No	No	No	No	No	No	No	No	177.9	28	24.60	25.70	No	91.00	5.00	No
7	76.00	3.00	5	30	39.00	4.30	Body/tail	Multilocular	Incidental	No	No	No	No	Yes	No	No	No	No	No	No	No	No	No		<10	19.10	26.30	No	114.00	4.00	No
8	71.00	3.00	3	47	46.00	22.10	Head/uncinate	Unilocular	Symptomatic	No	No	No	No	Yes	No	No	No	No	Yes	No	No	No	No	292.7	<10	29.60	21.50	No	77.00	3.00	No
9	85.00	5.00	8	35	45.00	10.80	Head/uncinate	Multilocular	Symptomatic	Yes	No	No	Yes	Yes	Yes	No	No	No	No	No	No	No	No	591.5	<10	16.20	6.60	Yes	300.00	24.00	No
10	83.00	4.00	8	42	36.00	14.00	Head/uncinate	Multilocular	Incidental	No	No	No	No	Yes	No	No	No	No	No	No	No	No	No	125.2	13	0.00	0.00	No	217.00	13.00	No
11	78.00	4.00	8	36	32.00	17.70	Head/uncinate	Unilocular	Incidental	No	No	No	No	Yes	No	No	No	No	Yes	No	No	No	No	110.9	<10	36.00	39.80	Yes	315.00	9.00	No
12	78.00	4.00	8	30	26.00	10.10	Body/tail	Multilocular	Incidental	No	No	No	No	No	No	No	No	No	No	No	No	No	No	401.3	<10	0.00	2.80	No	140.00	11.50	Yes
13	76.00	2.00	6	47	52.00	40.50	Head/uncinate	Unilocular	Incidental	No	No	No	No	Yes	No	No	No	No	Yes	No	No	No	No	1186.9	<10	56.00	45.00	No	464.50	13.50	Yes
14	75.00	2.00	9	52	50.00	14.80	Head/uncinate	Multilocular	Incidental	No	No	No	No	Yes	No	No	No	No	No	No	No	No	No	267.2	Not collected	24.00	35.00	No	218.50	20.00	Yes
15	77.00	3.00	4	40	35.00	9.10	Head/uncinate	Multilocular	Incidental	No	No	No	No	Yes	No	No	No	No	Yes	No	No	No	No	129.0	22	38.30	0.00	No	123.00	9.00	No
16	77.00	5.00	5	50	51.00	34.40	Head/uncinate	Multilocular	Incidental	No	No	No	No	Yes	No	No	No	No	Yes	No	No	No	No	88.5	Not collected	20.50	3.70	No	174.00	17.00	No
17	83.00	4.00	7	35	37.00	12.00	Head/uncinate	Multilocular	Incidental	No	No	No	No	Yes	No	No	No	No	No	No	Yes	No	Yes	Not collected	Not collected	0.00	4.30	No	274.00	16.00	No
18	75.00	3.00	5	69	66.00	127.60	Head/uncinate	Unilocular	Incidental	No	No	No	No	Yes	No	No	No	No	Yes	No	No	No	No	497.9	12	0.00	0.00	No	356.00	25.00	Yes
19	77.00	3.00	5	42	38.00	18.90	Head/uncinate	Multilocular	Incidental	No	No	No	No	Yes	No	Yes	No	No	Yes	No	No	No	No	39.4	< 10	49.90	38.90	No	266.00	17.00	Yes
20	75.00	5.00	6	46	48.00	41.90	Body/tail	Unilocular	Incidental	No	No	No	No	Yes	No	No	No	No	Yes	No	No	No	No	93.8	< 10	49.10	0.00	No	743.00	18.00	No
21	75.00	5.00	6	48	35.00	17.10	Head/uncinate	Multilocular	Incidental	No	No	No	No	Yes	No	No	No	No	Yes	No	No	No	No			24.30	0.00	No	226.00	17.00	No
22	67.00	2.00	5	95	97.00	306.00	Head/uncinate	Multilocular	Incidental	No	No	No	No	Yes	No	No	No	No	No	No	No	No	No	192.7	< 10	0.00	53.00	No	860.00	20.70	Yes
23	67.00	2.00	5	72	79.00	194.10	Head/uncinate	Multilocular	Incidental	No	No	No	No	Yes	No	No	No	No	Yes	No	No	No	No		81	44.20	36.60	No	180.00	27.00	No
24	67.00	2.00	5	58	69.00	71.70	Body/tail	Multilocular	Incidental	No	No	No	No	Yes	No	No	No	No	No	No	No	No	No		98	45.50	56.70	No	807.00	25.00	No
25	82.00	2.00	7	33	41.00	27.30	Head/uncinate	Multilocular	Incidental	No	No	No	No	Yes	No	No	No	No	Yes	No	No	No	No	5.0	<10	0.00	0.00	No	324.00	12.00	No
26	72.00	3.00	5	44	30.00	10.40	Body/tail	Multilocular	Incidental	No	No	No	No	Yes	Yes	Yes	No	Yes	Yes	No	No	No	No	557.6	< 10	25.30	0.00	No	301.00	28.00	No
27	64.00	3.00	7	42	38.00	14.30	Body/tail	Unilocular	Incidental	No	No	No	No	Yes	No	No	No	No	Yes	No	No	No	No	181.9	< 10	54.90	0.00	No	744.00	19.00	No
28	66.00	3.00	4	40	29.00	12.20	Head/uncinate	Multilocular	Incidental	No	No	No	No	Yes	Yes	No	No	No	No	No	No	No	No	469.1	< 10	42.10	29.50	No	642.00	18.00	No
29	59.00	5.00	6	75	65.00	80.40	Body/tail	Multilocular	Symptomatic	No	Yes	No	No	Yes	Yes	No	No	No	Yes	No	No	Yes	No	926.5	107	2.00	0.00	No	335.50	30.00	Yes
30	58.00	3.00	3	45	59.00	46.70	Head/uncinate	Multilocular	Incidental	No	No	No	No	Yes	No	No	No	No	No	No	No	Yes	No	63.5	< 10	8.60	0.00	No	146.00	17.00	No

BD-IPMN, branch-duct intraductal papillary mucinous neoplasm; CEA, carcinoembryonic antigen; CFS, Clinical Frailty Scale (CFS); CCI, Charlson Comorbidity Index; EUS, endoscopic ultrasound; HGD, high-grade dysplasia; Ica, invasive carcinoma; MPD, main pancreatic duct; RFA, radiofrequency ablation; CA 19-9 carbohydrate antigen 19-9.
Variant allele fraction (VAF) is the proportion of DNA carrying a specific gene variant such as *KRAS* or GNAS, high-risk DNA mutations refer to genetic alterations associated with higher malignancy risk.

Supplementary Table 2 Outcomes of EUS-guided chemoablation in pancreatic cystic lesions.

Author, year	Study type	Agents employed (n = subjects)	Complete (CR), partial (PR), or no response (NR)
Oh et al., 2008 [1]	Prospective	Ethanol + paclitaxel (n = 14)	79% CR, 14% PR, 7% NR
Oh et al., 2009 [2]	Prospective	Ethanol + paclitaxel (n = 10)	60% CR, 20% PR, 20% NR
Oh et al., 2011 [3]	Prospective	Ethanol + paclitaxel (n = 47)	62% CR, 13% PR, 25% NR
Moyer et al., 2016 [4]	Prospective	80% ethanol → paclitaxel + gemcitabine (n = 4)	75% CR
		Saline → paclitaxel + gemcitabine (n = 6)	67% CR
Moyer et al., 2017 [5]	Prospective RCT	80% ethanol → paclitaxel + gemcitabine (n = 18)	61% CR, 22% PR, 17% NR
		Saline → paclitaxel + gemcitabine (n = 21)	67% CR, 14% PR, 19% NR
Choi et al., 2017 [6]	Prospective	Ethanol + paclitaxel (n = 164)	72.2% CR, 19.6% PR, 8.2% NR
Krishna et al., 2024 [7]	Prospective	Large surface area microparticle paclitaxel (n = 6)	66.6% CR, 33.4% PR

CR, complete response; EUS, endoscopic ultrasound; NR, no response; PR, partial response; RCT, randomized controlled trial.

References

1 *Oh H-C, Seo DW, Lee TY* et al. New treatment for cystic tumors of the pancreas: EUS-guided ethanol lavage with paclitaxel injection. Gastrointest Endosc 2008; 67: 636-642
2 *Oh H-C, Seo DW, Kim SC* et al. Septated cystic tumors of the pancreas: is it possible to treat them by endoscopic ultrasonography-guided intervention? Scand J Gastroenterol 2009; 44: 242-247
3 *Oh HC, Seo DW, Song TJ* et al. Endoscopic ultrasonography-guided ethanol lavage with paclitaxel injection treats patients with pancreatic cysts. Gastroenterology 2011; 140: 172-179
4 *Moyer MT, Dye CE, Sharzehi S* et al. Is alcohol required for effective pancreatic cyst ablation? The prospective randomized CHARM trial pilot study. Endosc Int Open 2016; 4: E603-E607
5 *Moyer MT, Sharzehi S, Mathew A* et al. The safety and efficacy of an alcohol-free pancreatic cyst ablation protocol. Gastroenterology 2017; 153: 1295-1303
6 *Choi J-H, Seo DW, Song TJ* et al. Long-term outcomes after endoscopic ultrasound-guided ablation of pancreatic cysts. Endoscopy 2017; 49: 866-873

7 *Krishna SG, Ardeshna DR, Shah ZK* et al. Intracystic injection of large surface area microparticle paclitaxel for chemoablation of intraductal papillary mucinous neoplasms: Insights from an expanded access protocol. Pancreatology 2024; 24: 289-297

Supplementary Table 3 Comparative analysis of endoscopic ultrasound-guided radiofrequency ablation (EUS-RFA) parameters in the management of pancreatic cystic lesions (PCLs) and branch duct intraductal papillary mucinous neoplasms (BD-IPMNs).

Study	Number of pancreatic cystic lesions (PCLs)	Mean cyst diameter	Power (watts)	Impedance limit (Ohms)	Applications per lesion
Pai et al. [1]	6 PCLs, BD-IPMN = 1	3.65 cm	5-25	Not specified	Median 4.5 (range 2-7)
Barthet et al. [2]	17 Mucinous PCLs, BD-IPMN = 16	2.9 cm	50	100	1-3
Younis et al. [3]	Mixed lesions = 12, BD-IPMN = 4	3.6 cm (median)	50	100	1-7
Current (ERASE) Study	30, all BD-IPMNs	4.6 cm (median 4.1 cm)	50	400	Mean 14 (range 2-30)

References

1 *Pai M, Habib N, Senturk H* et al. Endoscopic ultrasound guided radiofrequency ablation, for pancreatic cystic neoplasms and neuroendocrine tumors. World J Gastrointest Surg 2015; 7: 52

2 *Barthet M, Giovannini M, Gasmi M* et al. Long-term outcome after EUS-guided radiofrequency ablation: Prospective results in pancreatic neuroendocrine tumors and pancreatic cystic neoplasms. Endosc Int Open 2021; 9: E1178-E1185

3 *Younis F, Ben-Ami Shor D, Lubezky N* et al. Endoscopic ultrasound-guided radiofrequency ablation of premalignant pancreatic-cystic neoplasms and neuroendocrine tumors: prospective study. Europ J Gastroenterol Hepatol 2022; 34: 1111-1115

Supplementary Table 4 Discordance between volumetric and molecular responses in BD-IPMNs after EUS-RFA.

Response category	Number of BD-IPMNs	Volumetric response	Molecular (NGS) response
Index EUS-RFA	28	35.7% (10/28) showed suboptimal volume reduction (<50%)	87.5% (7/8) showed a molecular response*
Volumetric non-responders	10	30% (3/10) demonstrated cyst enlargement	66.7% (2/3) showed a molecular response within this subgroup
≥ 2 EUS-RFA treatments in BD-IPMNs without complete volumetric response	6	No complete volumetric responses observed	83.3% (5/6) showed a molecular response within this subgroup

*NGS follow-up was available for eight of 10 BD-IPMNs with < 50% volume reduction.
BD-IPMN, branch duct intraductal papillary mucinous neoplasm; EUS-RFA, endoscopic ultrasound-guided radiofrequency ablation; NGS, next-generation sequencing.
